# *In silico* analysis identifies neuropilin-1 as a potential therapeutic target for SARS-Cov-2 infected lung cancer patients

**DOI:** 10.18632/aging.203159

**Published:** 2021-06-24

**Authors:** Song Hu, Zheyu Hu, Jiajia Qin, Chuwen Lin, Xuan Jiang

**Affiliations:** 1 Molecular Cancer Research Center, School of Medicine, Sun Yat-Sen University, Shenzhen 518107, Guangdong, China

**Keywords:** SARS-CoV-2, NRP1, TMPRSS2, immune infiltration, miRNA, COVID-19

## Abstract

The severe acute respiratory syndrome coronavirus 2 (SARS-CoV-2) causes coronavirus disease 2019 (COVID-19), and is highly contagious and pathogenic. TMPRSS2 and Neuropilin-1, the key components that facilitate SARS-CoV-2 infection, are potential targets for treatment of COVID-19. Here we performed a comprehensive analysis on NRP1 and TMPRSS2 in lung to provide information for treating comorbidity of COVID-19 with lung cancer. NRP1 is widely expressed across all the human tissues while TMPRSS2 is expressed in a restricted pattern. High level of NRP1 associates with worse prognosis in multiple cancers, while high level of TMPRSS2 is associated with better survival of Lung Adenocarcinoma (LUAD). Moreover, NRP1 positively correlates with the oncogenic Cancer Associated Fibroblast (CAF), macrophage and endothelial cells infiltration, negatively correlates with infiltration of CD8^+^ T cell, the tumor killer cell in Lung Squamous cell carcinoma (LUSC). TMPRSS2 shows negative correlation with the oncogenic events in LUAD. RNA-seq data show that NRP1 level is slightly decreased in peripheral blood of ICU admitted COVID-19 patients, unaltered in lung, while TMPRSS2 level is significantly decreased in lung of COVID-19 patients. Our analysis suggests NRP1 as a potential therapeutic target, while sets an alert on targeting TMPRSS2 for treating comorbidity of COVID-19 and lung cancers.

## INTRODUCTION

Lung cancers, the most commonly diagnosed cancer in males and third in females, account for the highest number of cancer-related deaths in combined sexes globally [1]. The coronavirus disease-19 (COVID-19), caused by a newly identified virus SARS-CoV-2, has spread worldwide. SARS-CoV-2 attacks multiple human organs, especially the distal bronchiole and alveoli in lung [2], which overlaps with the affected organ in lung cancer. This ongoing pandemic has caused more than 1.76 million death globally and become the most emergent threat to public health. Because lung cancer patients bear greater predisposition to respiratory complications, comorbidity of COVID-19 in lung cancer patients leads to higher mortality rate and higher burden of severity [3, 4], which raises the need for a special regimen for COVID-19 patients with lung cancer.

NRP1 is a highly conserved single-pass plasma membrane receptor, which binds with Semaphorins and VEGF. Because of the interaction with VEGF, NRP1 enhances angiogenesis, including the tumor angiogenesis. NRP1 is found to be upregulated in several types of cancer and favors tumor cell growth, survival and metastasis [5]. In addition to Semaphorins and VEGF mediated signaling, NRP1 responds to multiple other pathways, including receptor tyrosine kinases, integrins, HGF, FGF, TGF-β, Galectin, etc, making it a signaling hub on the cell surface to integrate the outside stimuli into cells [5]. NRP1 is also a key immunoregulatory receptor, which stabilizes Treg cells, the most prevalent immunosuppressive cells, and prevents CD8^+^ T cells reinvigoration in response to checkpoint inhibitors [6]. Despite its significant role in immune system, the onco-immunity role of NRP1 in lung cancers has never been studied.

TMPRSS2 codes for a transmembrane serine protease, which can stimulate a proteolytic cascade to promote degradation of extracellular matrix and the cell invasion of prostate cancer [7]. Moreover, TMPRSS2 tends to form a fusion with ERG, which is present in 40-80% prostate cancers and promotes cell migration and metastasis of prostate cancer [8, 9]. However, its role in lung cancers is rarely studied.

ACE2, angiotensin-converting enzyme 2, converts angiotensin II to angiotensin-(1–7), which induces the release of vasodilators and plays a protective role in cardiovascular disease and diabetes [10]. Moreover, ACE2 is the membrane bound receptor for several coronaviruses, including SARS-CoV-2 [11]. NRP1, binding to furin-cleaved substrates, together with the S protein priming agent TMPRSS2, facilitates SARS-CoV-2 cell entry and potentiates its infectivity [11, 12]. Due to the roles in SARS-CoV-2 infection, ACE2, NRP1 and TMPRSS2 are targets for drug design for COVID-19. However, the impact of targeting those molecules on lung cancers hasn’t been studied, which makes the outcome of this treatment in COVID-19 patients with lung cancer ambiguous.

In this study, we conducted a systemic *in silico* analysis on NRP1, TMPRSS2 in LUSC and LUAD with online databases. NRP1 is widely expressed across multiple organs, while TMPRSS2 is expressed in a restricted pattern. Both are decreased in human lung cancer tissues, consistent with the increased methylation in promoter area and increased microRNAs that target NRP1 or TMPRSS2 in lung cancer tissues. LUSC patients with high NRP1 level have poor prognosis, while LUAD patients with high TMPRSS2 level show better survival rate. Consistent with the association between gene abundance and survival curve, NRP1 is positively correlated with pro-tumorigenic immune cells and genes in cancer tissues in LUSC, while TMPRSS2 shows no correlation or negatively associated with the factors that may facilitate cancer progression in LUAD. Finally, the expression level of NRP1 and TMPRSS2 in COVID-19 patients was analyzed using the public datasets: NRP1 is decreased in peripheral blood of COVID-19 patients admitted to intensive care unit (ICU), but the level of NRP1 in the lung of COVID-19 patients is not changed. TMPRSS2 is significantly decreased in the lung of COVID-19 patients. Taken altogether, NRP1 might be a potential target for comorbidity of COVID-19 and lung cancer, although further experimental validation is lacking. However, TMPRSS2 should not be targeted in lung cancer patients infected with SARS-CoV-2.

## RESULTS

### The expression level of NRP1, TMPRSS2 and ACE2 in human normal tissues

**Figure 1:**
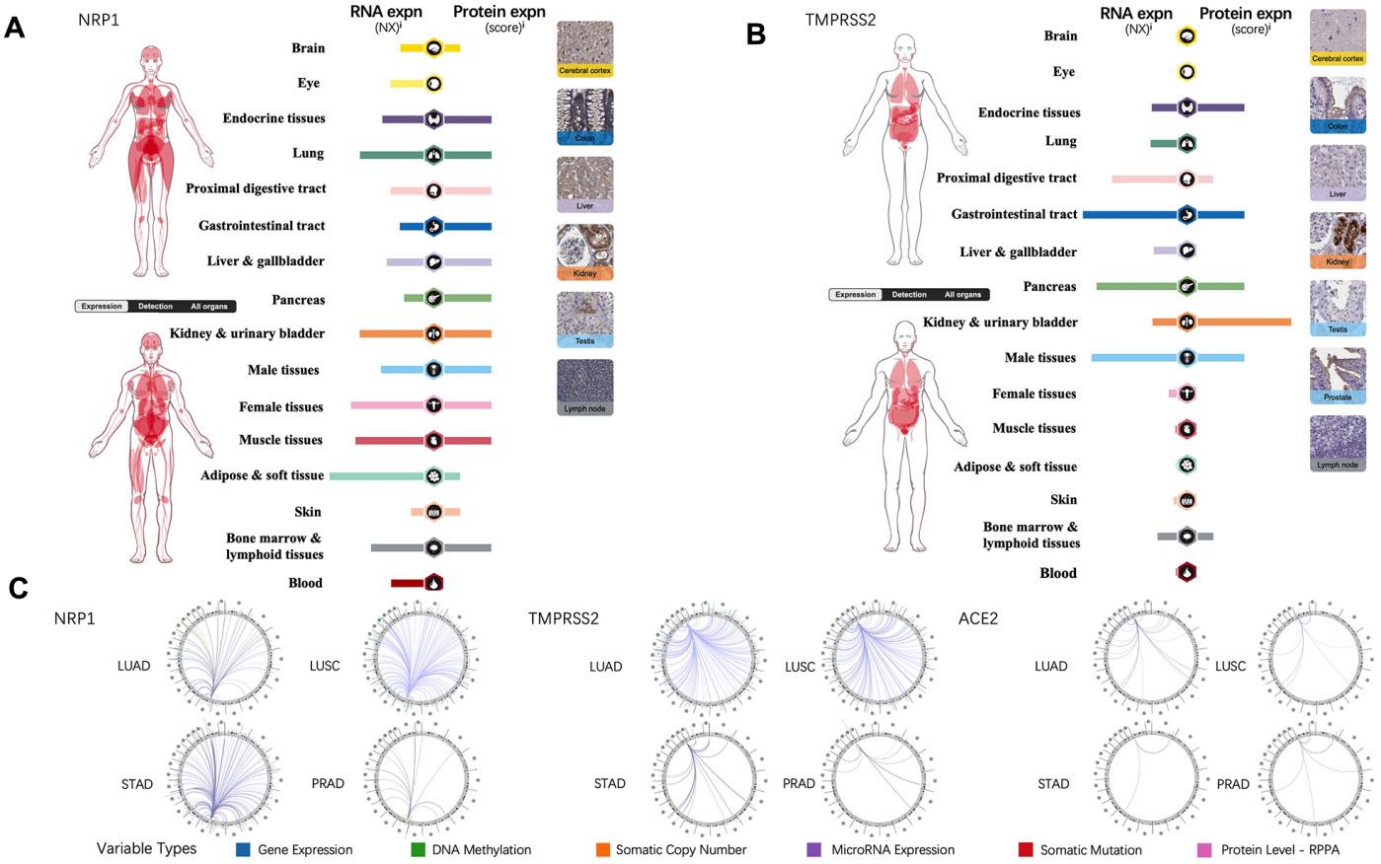
**NRP1, TMPRSS2 and ACE2 expression in human normal tissue and their interrelation with other genes.** (**A**) The expression levels of NRP1 in human tissues. (**B**) The expression levels of TMPRSS2 in human tissues. (**C**) The correlation between certain genes and NRP1, TMPRSS2, ACE2 in LUAD, LUSC, STAD and PRAD.

Tissue-specific expression profiling of NRP1, TMPRSS2 and ACE2 were analyzed through online database, the Human Protein Atlas, which provides expression information based on immunohistochemistry and high-throughput mRNA sequencing [13]. In contrast to TMPRSS2 and ACE2, mRNA of NRP1 is present in all the organs and protein of NRP1 can be detected in every organ except for eye and blood (Figure 1A). TMPRSS2 is expressed in gastrointestinal tract, endocrine tissues, kidney, urinary bladder, male tissues, bone marrow and lymphoid tissues (Figure 1B), while ACE2 expression is restricted to gastrointestinal tract, kidney, urinary bladder, male tissues, liver and gallbladder [14]. To further explore the association of NRP1, TEMPRSS2 and ACE2 with other genes in cancers, the Regulome Explorer is used to visualize the multitude interaction of these genes with other factors within the context of LUAD, LUSC, STAD and PRAD. As shown in Figure 1C, ACE2 barely shows any interaction in LUAD, LUSC, STAD or PRAD, but NRP1 and TMPRSS2 have multiple interactions in LUAD and LUSC, indicating significant roles of NRP1 and TEMPRSS2 in lung cancers.

### NRP1 and TMPRSS2 expression analysis in lung cancers

**Figure 2:**
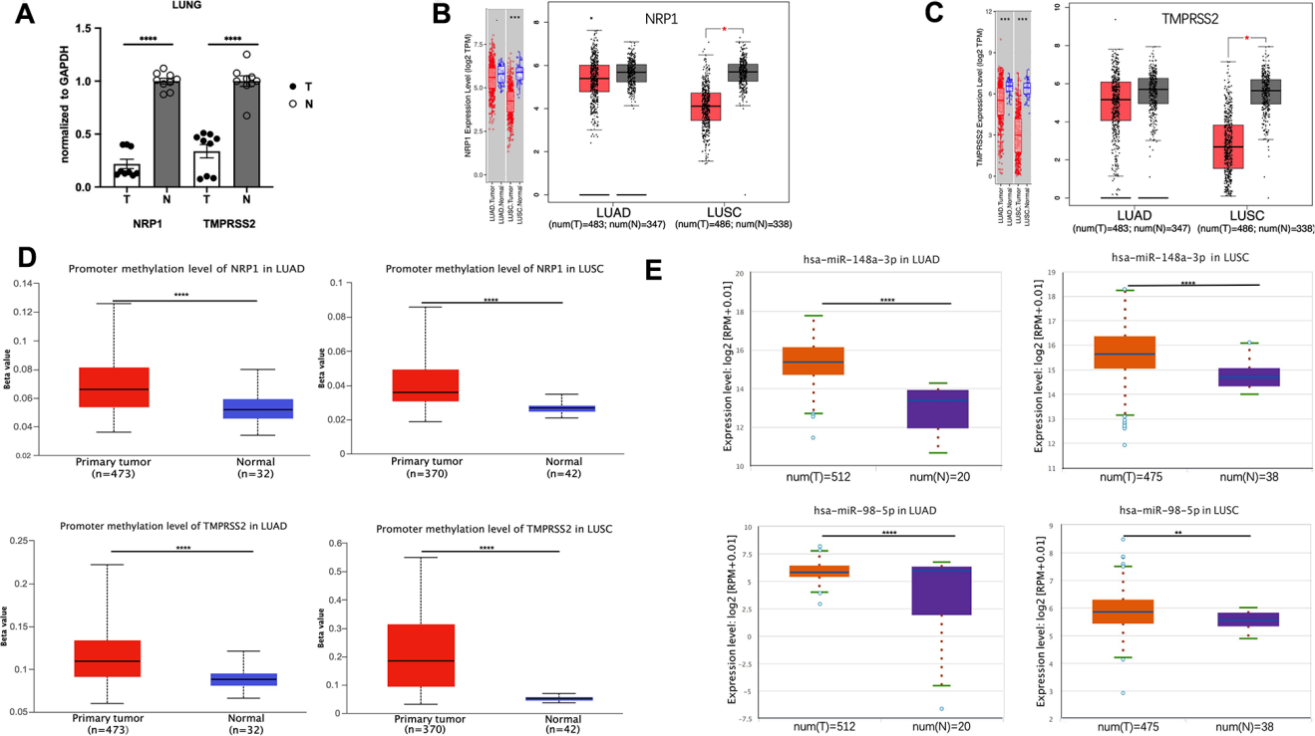
**The promoter methylation level of NRP1(TMPRESS2) and miRNA expression level in lung tumors.** (**A**) qPCR analysis of the expression of NRP1 and TMPRSS2 in LUAD tissues and paired normal tissues. n=3, **** P<0.0001. (**B**) The expression of NRP1 analyzed with TIMER and GEPIA. (**C**) The expression of TMPRSS2 analyzed with TIMER and GEPIA. (**D**)The promoter methylation level of NRP1 and TMPRSS2 in LUAD or LUSC analyzed with UALCAN. (**E**) The hsa-mir-148a and hsa-mir-98-5p expression level in LUAD and LUSC analyzed with ENCORI.

ACE2 expression level is hardly detective in peripheral blood or lung compared with NRP1 and TMPRSS2 (Figure 1A, 1B), and ACE2 downregulation after viral infection increases the severity of COVID-19 disease [15]. These two effects make ACE2 not an ideal target for treating COVID-19 disease, so we focus on NRP1 and TMPRSS2. To better understand the role of NRP1 and TMPRSS2 in lung cancers, we analyzed their expression levels in tumors with web server TIMER and GEPIA, which utilize data from The Cancer Genome Atlas Program (TCGA), a comprehensive database containing genomic, epigenomic, transcriptomic, and proteomic data of both tumor tissues and the paired normal tissues. Both NRP1 and TMPRSS2 are decreased in tumor tissues of LUAD and LUSC compared with paired normal tissues (Figure 2B, 2C). We further validated the expression in LUAD tumors with real-time PCR. (Figure 2A).

In the mammalian genome, DNA methylation is an epigenetic modification by which methyl groups are added to DNA, resulting in gene transcription repression [16]. DNA methylation pattern is usually abnormal in tumor tissues, and thus can be used as a prognostic biomarker [17–19]. Promoter methylation level of NRP1 and TMPRSS2 promoter in LUAD and LUSC was analyzed with UALCAN web tool. Compared with normal tissues, NRP1 and TMPRSS2 promoters in LUAD and LUSC both show increased methylation (Figure 2D), consistent with the decreased NRP1 and TMPRSS2 expression in tumor tissues.

MicroRNA, a 19-22 nucleotide small non-coding RNA, can bind to the 3’UTR of targeted mRNA which leads to a mRNA degradation or translation inhibition [20]. hsa-mir-148a-3p is reported to suppress angiogenesis through targeting NRP1 [21]. hsa-mir-98-5p is reported to repress TMPRSS2 in endothelial cells [22]. ENCORI was used to analyze the expression level of hsa-mir-148a-3p and has-mir-98-5p. Both are increased in LUAD and LUSC tumor tissues compared with paired normal tissues (Figure 2E). The level of pre-mir-148a and pre-mir-98 were further analyzed in tumor tissues. Consistently, both pre-miRs are increased in LUAD tumors (Supplementary Figure 1A). The elevated miRNAs and increased promoter methylation provide a strong rationale for the decreased NRP1 and TMPRSS2 level in LUAD and LUSC tumors.

### Prognostic impact of NRP1 and TMPRSS2 expression in cancer patients

**Figure 3:**
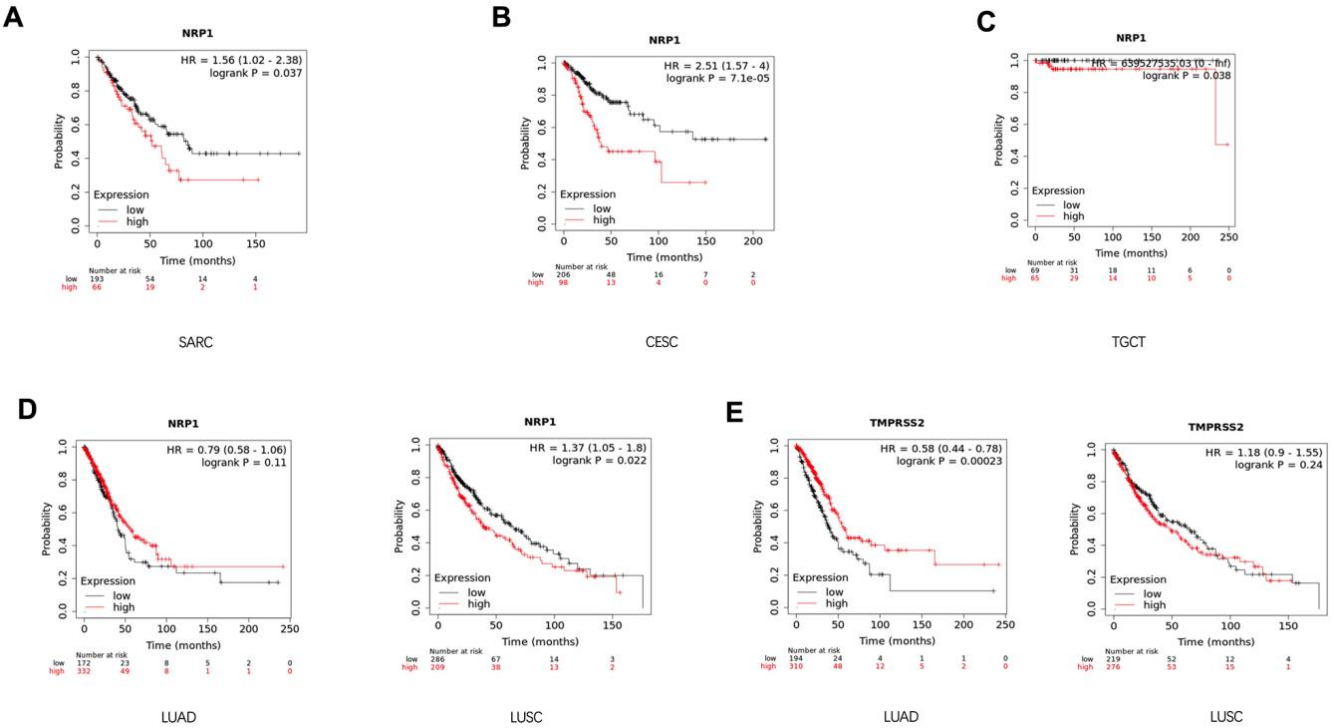
**Survival analysis of cancer patients with differential gene expression level.** (**A**–**D**) Survival curve of SARC (n=259), CESC (n=304), TGCT (n=134), LUSC (n=501) and LUAD (n=513) patients with differential level of NRP1 expression. (**E**) Survival curve of LUSC and LUAD patients with differential level of TMPRSS2 expression. Analysis was done with Kaplan-Meier Plotter.

In attempts to gain better insights into the role of NRP1 and TMPRSS1 in cancers, the survival curve of cancer patients with differential expression level of NRP1 or TMPRSS2 was analyzed with the Kalpan-Meier plotter, a large database combining information from GEO, EGA and TCGA. High NRP1 expression correlates with reduced overall survival for sarcoma (SARC) (Figure 3A), CESC (Figure 3B), Testicular Germ Cell Tumors (TGCT) (Figure 3C) and LUSC (Figure 3D) patients in Kaplan-Meier databases, while high TMPRSS2 mRNA in tumors associates with increased survival and better prognosis for LUAD (Figure 3E) patients. However, the expression level of NRP1 had no differential impact on LUAD (Figure 3D) patient’s survival, and the expression of TMPRSS2 had no differential impact on the survival of LUSC (Figure 3E) patients. The prognostic relationship between NRP1 and TMPRSS2 expression level and cancers were further confirmed by analysis with GEPIA (Supplementary Figure 2).

Consistent with the result that low level of NRP1 associates with better prognosis, high level of miR-148a associates with better clinical outcome in multiple cancers, including LUAD (Supplementary Figure 1B), Liver Hepatocellular Carcinoma (LIHC) (Supplementary Figure 3A), OV (Supplementary Figure 3B), Cervical and Endocervical Cancer (CESC) (Supplementary Figure 3C) and UCEC (Supplementary Figure 3D) in the analysis by the Kaplan-Meier plotter. This association was further confirmed with GEPIA (Supplementary Figure 1B, Supplementary Figure 3E–3H). The level of miR-148a doesn’t show association with the prognosis of LUSC, while high level of miR-98 correlates better prognosis with LUSC (Supplementary Figure 1C–1E).

### Correlation between NRP1 Expression and tumor immune infiltration

Immune cell infiltration in tumors correlates with the development of cancers and is of prognostic value in multiple types of tumors, including lung cancers [23–25]. To this end, TIMER was applied to investigate the association between immune infiltration and expression level of NRP1 and TMPRSS2 in LUSC and LUAD.

**Figure 4:**
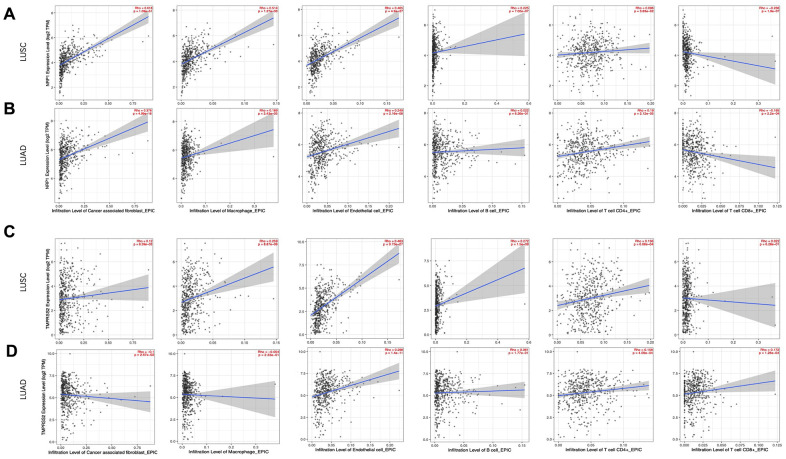
**Correlation analysis between NRP1/TMPRSS2 expression and tumor immune infiltration.** (**A**, **B**) Correlation analysis between NRP1 expression and tumor immune infiltration in LUSC (n=501) and LUAD (n=515). (**C**, **D**) Correlation analysis between TMPRESS2 expression and tumor immune infiltration in LUSC and LUAD. None purity-adjusted for all the panels.

Cancer associated fibroblast (CAF), Macrophage and Endothelial cells are usually considered to be pro-tumorigenic, favoring tumor angiogenesis, metastasis and chemotherapy resistance, although they demonstrate great heterogeneity and diverse functions [26–29]. NRP1 expression level is positively correlated with infiltration of CAFs (r=0.618, p<0.001), Macrophage (r=0.514, p<0.001) and Endothelial cell (r=0.465, p<0.001) (Figure 4A). CD8^+^ and CD4^+^ T cells are cytotoxic immune cells that directly or indirectly kill cancer cells [30, 31]. NPR1 amount was negatively correlated with infiltration of CD8^+^ T cell (r= -0.236, p<0.001) in LUSC (Figure 4A), while TMPRSS2 expression level was positively correlated with infiltration of CD8^+^ T cell (r= 0.172, p<0.001) and CD4^+^ T cell (r=0.158, p<0.001) in LUAD (Figure 4D). Consistent with the increased survival in TMPRSS2 high LUAD patients, TMPRSS2 negatively correlated with infiltration level of CAFs (r=-0.1, p<0.05) and in LUAD (Figure 4D). The role of B cells in tumor immune response are ambiguous, it is reported to regulate anti-tumor T-cell response both positively and negatively [32]. NRP1 dosage positively associates with B cell infiltration in LUSC (Figure 4A), while TMPRSS2 dosage doesn’t show any significant correlation with B cell infiltration in LUAD (Figure 4D).

**Figure 5:**
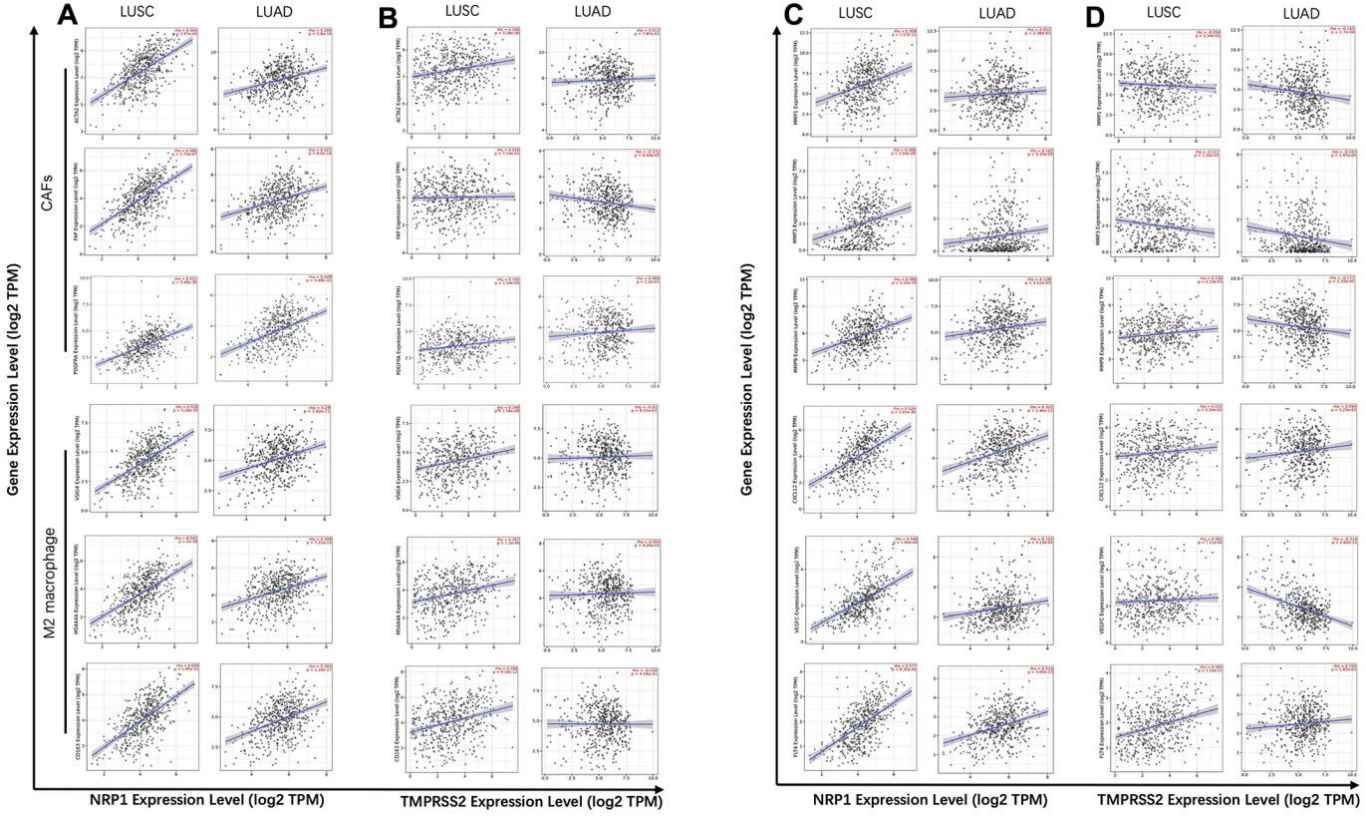
**Correlation analysis between NRP1/TMPRSS2 and pro-tumorigenic factors.** (**A**) Correlation analysis between NRP1 and markers for CAFs and M2 macrophage in LUSC (n=501) and LUAD (n=515). (**B**) Correlation analysis between TMPRSS2 and markers for CAFs and M2 macrophage in LUSC and LUAD. (**C**) Correlation analysis between NRP1 and MMP1, MMP3, MMP9, VEGFC, FLT4 and CXCL12 in LUSC and LUAD. (**D**) Correlation analysis between TMPRSS2 and MMP1, MMP3, MMP9, VEGFC, FLT4 and CXCL12 in LUSC and LUAD. None purity-adjusted for all the panels.

To gain insights into the mechanism of high NRP1 level and low TMPRSS2 level associating with worse prognosis in lung cancers, specific molecules were analyzed to further dissect the immune infiltration correlating with the expression of NRP1 or TMPRSS2. M2 macrophage polarization usually promotes tumor angiogenesis, growth and metastasis [33, 34]. TIMER database was used to explore the relationship between the expression of NRP1 and TMPRSS2 with ACTA2, FAP, PDGFRA, the markers for CAF and VSIG4, CD163, MS4A4A, the markers for M2 macrophage [35, 36]. NRP1 expression level shows stronger association with CAF and M2 macrophage markers in LUSC compared with those in LUAD (Figure 5A, Supplementary Figure 4), which is consistent with the decreased survival rate in high NRP1 LUSC patients (Figure 3D). TMPRSS2 expression level is negatively correlated with CAF marker FAP in LUAD, but no significant correlation with other marker genes (Figure 5B, Supplementary Figure 4).

Matrix metalloproteinases (MMPs) are a group of endopeptidases that can degrade extracellular matrix to facilitate tumor invasion and are also involved in angiogenesis to promote cancer cell growth and migration [37]. VEGFC and its receptor FLT4 has pro-tumorigenic function by promoting lymphangiogenesis. CXCL12 also plays a role to initiate and promote tumor through CXCL12/CXCR4 axis [38]. Analysis with TIMER showed strong correlation between NRP1 expression and the abundance of tumor progression-related genes, MMP1, MMP3, MMP9, VEGFC, FLT4 and CXCL12 in LUSC, while there isn’t clear association between NRP1 expression and this panel of pro-tumorigenic genes in LUAD (Figure 5C, Supplementary Figure 5). TMPRSS2 shows no correlation or even negative correlation with this panel of genes in LUSC or LUAD (Figure 5D, Supplementary Figure 5).

### SARS-CoV-2 infection is likely to decrease NRP1 and TMPRSS2 expression

**Figure 6:**
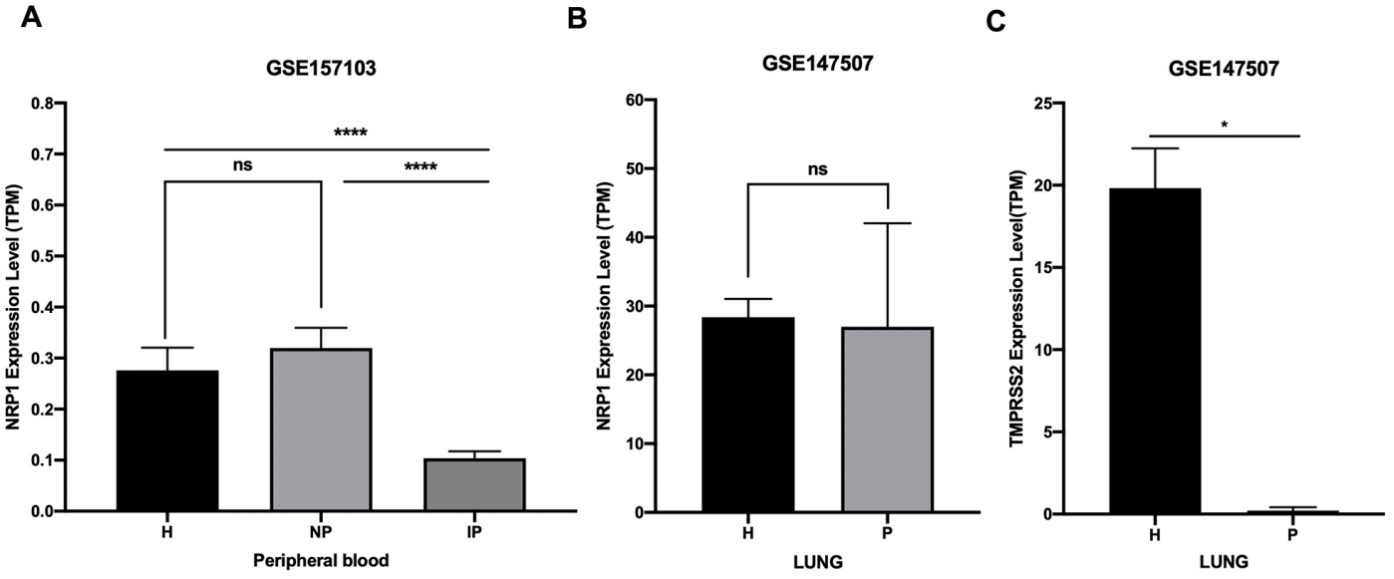
**Expression analysis of NRP1 and TMPRSS2 after SARS-CoV-2 infection.** (**A**) TPM of NRP1 in peripheral blood RNA seq data. H: healthy control. NP: Non-ICU patients with COVID-19. IP: ICU patients with COVID-19. (**B**) TPM of NRP1 in lung RNA seq data. H: healthy control. P: COVID-19 patients. (**C**) TPM of TMPRSS2 in lung RNA seq data. H: healthy control. P: COVID-19 patients. ****, P<0.0001. *, P<0.05. ns, not significant.

NRP1 and TMPRSS2 facilitate the entry into host cell of SARS-CoV-2 [12]. To prevent the spreading of SARS-CoV-2 *in vivo*, it is necessary to understand their expression kinetics after SARS-CoV-2 infection. Online public data (GSE157103 and GSE147507) of RNA-seq with peripheral blood and lung samples from COVID-19 patients and health controls were subjected to analysis. Results show that the expression of NRP1 is decreased in peripheral blood of COVID-19 patients who are admitted into intensive care unit (ICU), but not in non-ICU COVID-19 patients (Figure 6A). Moreover, NRP1 is not decreased in the lung tissue of COVID-19 patients (Figure 6B). TMPRSS2 is reduced in the lung of COVID-19 patients (Figure 6C).

## DISCUSSION

SARS-CoV-2, being highly transmissible and pathogenic, has spread world-wide and become the most prevalent public health emergency. Patients who need to visit hospitals are at higher risk of infection. Lung cancer patients usually have airway obstruction which enhances the severity of clinical manifestation in comorbidity of COVID-19 with lung cancer. Lung cancer patients bear compromised immunity, exerting the patients further susceptibility to virus, while the fragile immune system in patients is further disrupted by SARS-CoV-2, making the poor prognosis of comorbidity of COVID-19 with lung cancer [39]. NRP1 and TMPRSS2 are key entry elements and therapeutic targets for SARS-CoV-2. Our work makes a comprehensive evaluation of NRP1 and TMPRSS2 in lung cancers, providing information for drug development and support for COVID-19 patients with lung cancers.

Our analysis indicates that high level of NRP1 is associated with poor survival of multiple cancers, including SARC, CESC, TGCT and LUSC, while high level of TMPRSS2 is associated with better prognosis in LUAD. Expression level of NRP1 is positively correlated with oncogenic immune cells and genes in cancer tissues in LUSC, suggesting NRP1 might be a pro-tumorigenic factor and potential therapeutic target in LUSC. So targeting the host factor NRP1 in COVID-19 patients with LUSC may suppress both the transmission of NRP1 and the tumor development. However, further experimental validation is necessary to confirm the pro-tumorigenic role of NRP1 in LUSC. TMPRSS2 shows no correlation or negative correlation with the factors that facilitate cancer progression in LUAD, setting an alert on targeting TMPRSS2 in comorbidity of COVID-19 and lung cancer.

NRP1 is decreased, but still quite abundant, in the peripheral blood of ICU COVID-19 patients (Figure 6A), and not altered in lung of COVID-19 patients (Figure 6B) compared with controls. The abundant level of NRP1 in COVID-19 patients makes it likely to get good response to the targeting strategy in patients.

The association of high level of miR-148a-3p with better prognosis in multiple cancers might be due to repression of NRP1, which promotes cancer cell growth, survival, migration, and angiogenesis within tumors [5]. Therefore, miR-148a-3p mimic is a potential therapeutic approach for those cancers. In a small RNA sequencing analysis of peripheral blood by another group, miR-148a-3p was significantly decreased in COVID-19 patients compared with healthy controls [40]. In current analysis, NRP1 is a candidate therapeutic target for treating COVID-19 patients with LUSC. The decrease of miR-148a-3p in COVID-19 patients further rationalizes miR-148a-3p mimic as an ideal candidate drug for comorbidity of COVID-19 and LUSC.

ACE2 deficiency is shown to increase the severity of SARS-CoV-2 infection due to the inflammation inhibitory effect of ACE2 via degrading angiotensin II [15]. The entry of SARS-CoV-2 markedly down-regulates ACE2 in the host cells, subjecting the patients with ACE2 deficiency to increased risk of inflammation and thrombosis [15]. Therefore, targeting ACE2 as an antiviral therapy is still controversial, although the ACE2 can be used as the prophylactic target before the entry of SARS-CoV-2 virus into humans. High level of ACE2 associates with better prognosis in LUSC [41], indicating potential risk of targeting ACE2 in comorbidity of COVID-19 and LUSC. Although respiratory tract is the primary target of SARS-CoV-2, accumulated evidence verifies central nervous system (CNS) as an affected organ of SARS-CoV-2 infection [42]. NRP1 is abundant in the central nervous system (Figure 1A) especially in olfactory bulb, which potentiates the infection of CNS through entry of the olfactory epithelial cells in nasal cavity [12]. The abundance of NRP1 in CNS makes it a candidate target to prevent CNS infection and disease manifestations of COVID-19. Combining our analysis, NRP1 is a potential therapeutic target for treating comorbidity of COVID-19 and lung cancers.

## CONCLUSIONS

To sum up, NRP1 is widely expressed across multiple tissues while TMPRSS2 is expressed in a restricted pattern. High level of NRP1 in lung cancer tissues is associated with poor survival, making it a potential therapeutic target in COVID-19 patients with lung cancer, while high level of TMPRSS2 correlates with better prognosis, excluding the attempt of targeting TMPRSS2 in comorbidity. NRP1 level is mildly decreased, but still quite abundant in peripheral blood of ICU admitted COVID-19 patients, while not changed in lung of COVID-19 patients compared with health control. Combing the analysis in lung cancer and COVID-19, NRP1 is a potential therapeutic target for treating comorbidity of COVID-19 with lung cancers. Moreover, the abundance of NRP1 in COVID-19 patients makes it feasible to target NRP1 and get response in patients.

## MATERIALS AND METHODS

### Ethical statement concerning the use of human objects

The human LUAD tumor tissues were obtained from patients in Guangzhou First People’s Hospital. Informed consents were obtained from all individuals.

### Expression and survival analysis

Expression level of ACE2, NRP1 and TMPRSS2 in different tissues was analyzed with The Human Protein Atlas [43] (https://www.proteinatlas.org/). Expression level of NRP1, TMPRSS2 in different tumors and paired controls is analyzed with GEPIA (http://gepia.cancer-pku.cn/) and Diff Exp module in TIMER database (https://cistrome.shinyapps.io/timer/). Expression level of hsa-miR-148a-3p, hsa-miR-98-5p is analyzed with the Pan-Cancer module in ENCORI [44] (http://starbase.sysu.edu.cn/index.php), which integrates expression profiles of both non-coding RNA and all protein-coding genes from TCGA.

Survival curve of cancer patients with differential level of NRP1, TMPRSS2, is analyzed with the Kaplan-Meier Plotter [45] (https://kmplot.com/analysis/) and GEPIA database. Survival curve of lung cancer patients with differential level of hsa-miR-148a-3p and hsa-miR-98-5p is analyzed with Kaplan-Meier Plotter and Pan-Cancer module of ENCORI.

### Integrative data visualization

The expression of NRP1, TMPRSS2 and its correlation with other genes in tumors were integratively visualized as a circus plot using the web tool Cancer Regulome (http://explorer.cancerregulome.org/). Pairwise correlation was analyzed with the Spearman’s rank order correlation. Only the genes with -log (p)≥ 6 were shown in the plot.

### Methylation analysis

Methylation level of NRP1 and TMPRSS2 promoter in lung tumor tissues was analyzed with UALCAN database, which is a comprehensive and interactive web tool to perform multiple in-depth analysis on the TCGA data [46].

### Immune infiltration analysis

The correlation between NRP1, TMPRSS2 expression and the infiltration level of immune cells or the levels of several pro-tumorigenic genes were analyzed with Gene module in TIMER web server (https://cistrome.shinyapps.io/timer/).

### Expression analysis with RNA seq data

The expression level of NRP1 and TMPRSS2 in COVID-19 patients was analyzed with RNA seq data deposited online. Expression level of NRP1 in peripheral blood from health control and ICU admitted COVID-19 patients or none-ICU COVID-19 patients was analyzed with data GSE157103. Expression level of TMPRSS2 or NRP1 from lung of health control or COVID-19 patients was analyzed with data GSE147507.

### Quantitative reverse transcriptase-polymerase chain reaction (RT-PCR) analysis

RNA from three LUAD tumor tissues and paired normal tissues were prepared with Trizol (ThermoFisher, cat. #15596026). 1μg RNA was reverse transcribed into cDNA with the PrimeScript® RT reagent Kit with gDNA Eraser (TaKaRa, cat. #RR047A). cDNA was 100-times diluted and RT-PCR reactions were then performed in triplicates using TB Green Premix Ex Taq II (TaKaRa, cat. #RR820A). Primer sequence is as follows:

hGAPDH-F: GTCTCCTCTGACTTCAACAGCG;

hGAPDH-R: ACCACCCTGTTGCTGTAGCCAA;

hNRP1-F: AACAACGGCTCGGACTGGAAGA;

hNRP1-R: GGTAGATCCTGATGAATCGCGTG;

hTMPRSS2-F: CCTCTAACTGGTGTGATGGCGT;

hTMPRSS2-R: TGCCAGGACTTCCTCTGAGATG;

U6-Control-F: CTCGCTTCGGCAGCACA

U6-Control-R: AACGCTTCACGAATTTGCGT

hsa_pre_mir98_F: TTCTGCTCATGCCAGGGTG

hsa_pre_mir98_R: ACCAGGGAAAGTAGTAAGTTG

hsa_pre_mir148a_F: GAGGCAAAGTTCTGAGACAC

hsa_pre_mir148a_R: GTTCTGTAGTGCACTGAC

### Statistical analysis

Graphs were generated with GraphPad PRISM software. Statistical significance was calculated in R version 3.2.3 by Student’s t test. The null hypothesis of the medians/means being equal was rejected at α = 0.05 and p values were generated by unpaired Student’s t test and presented in figures.

## Supplementary Material

Supplementary Figures
