# Supplementary material for: In silico analysis identifies neuropilin-1 as a potential therapeutic target for SARS-Cov-2 infected lung cancer patients
Source: Aging (Albany NY). 2021 Jun 24;13(12):15770–84. doi: 10.18632/aging.203159 (PMC8266340; doi:10.18632/aging.203159)
Supplement: Supplementary Figures [file aging-13-203159-s001.pdf]

SUPPLEMENTARY FIGURES

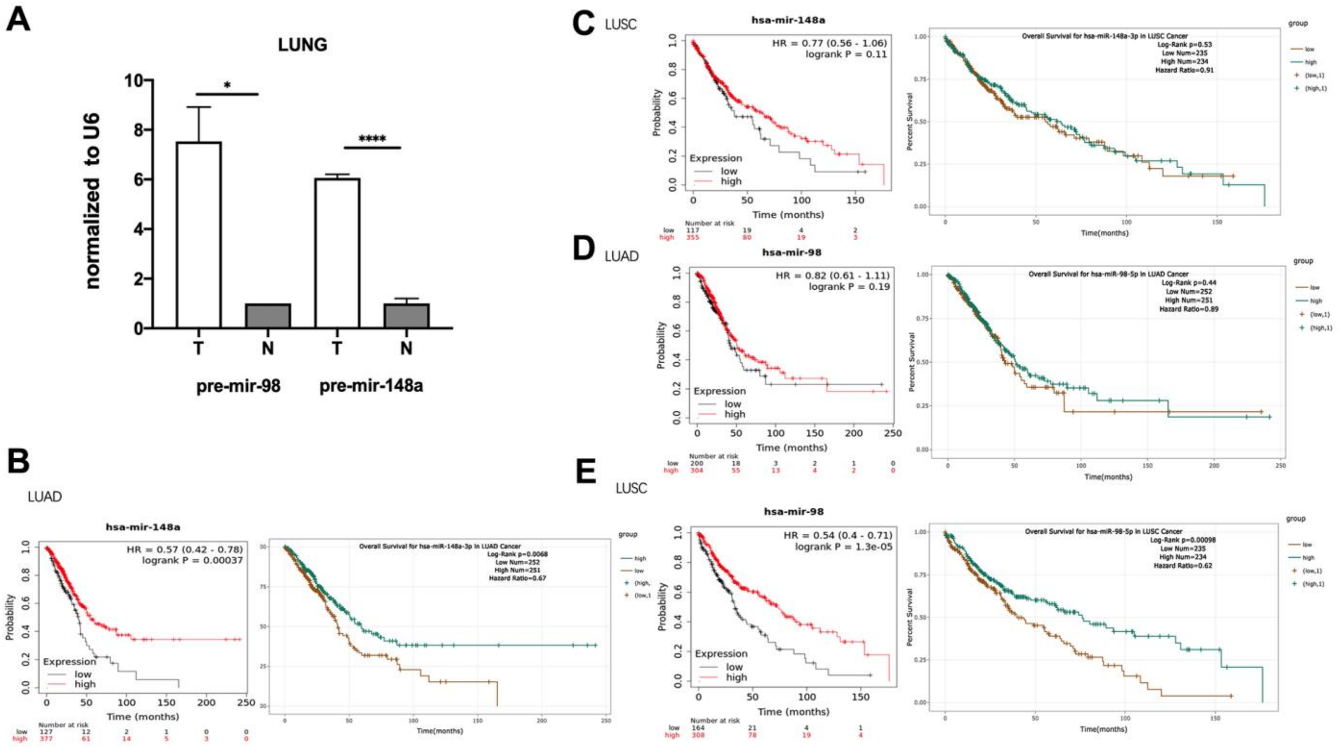

Supplementary Figure 1. Survival analysis of lung cancer patients with differential microRNA expression level. (**A**) qPCR analysis of pre-mir-98 and pre-miR-148a in LUAD tissues and paired normal tissues. n=3, * P<0.05, **** P<0.0001. (**B**, **C**) Survival curve of LUSC and LUAD patients with differential level of miR-148a expression. (**D**, **E**) Survival curve of LUSC and LUAD patients with differential level of miR-98 expression.

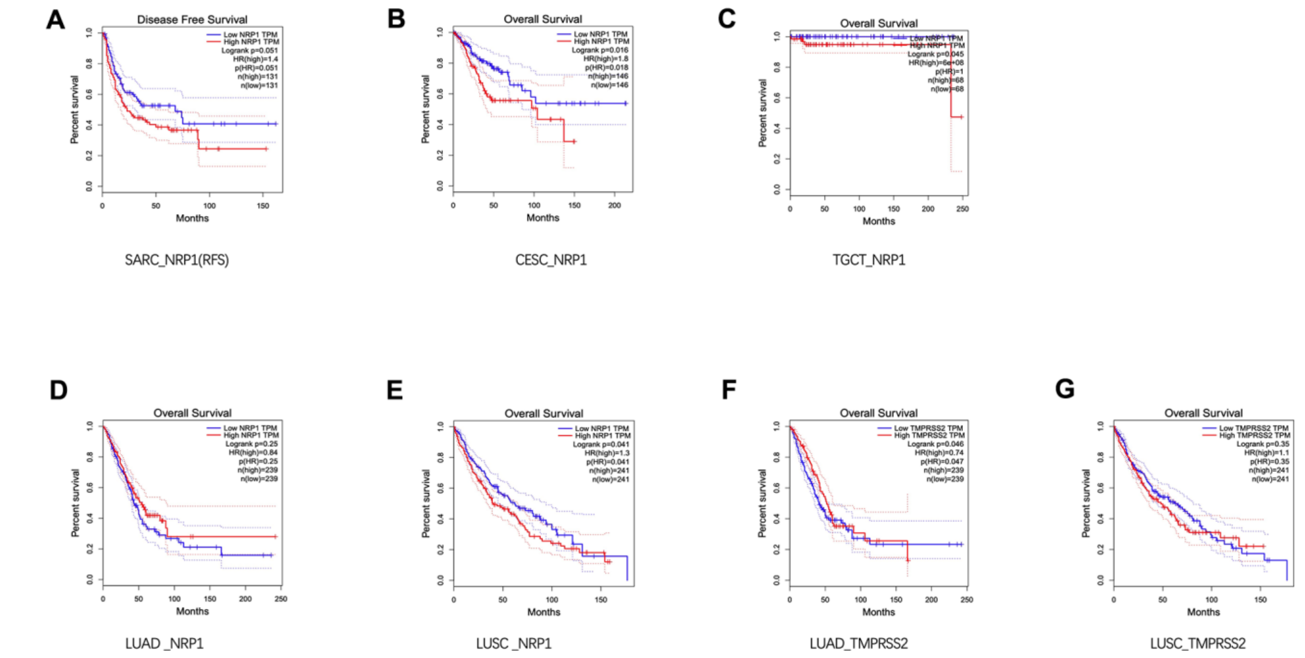

Supplementary Figure 2. Survival analysis of cancer patients with differential NRP1 or TMPRSS2 level. Survival curve of SARC (**A**), CESC (**B**), TGCT (**C**), LUAD (**D**) and LUSC (**E**) patients with different level of NRP1. Survival curve of LUAD (**F**), LUSC (**G**) patients with different level of TMPRSS2. Analysis was done by GEPIA database.

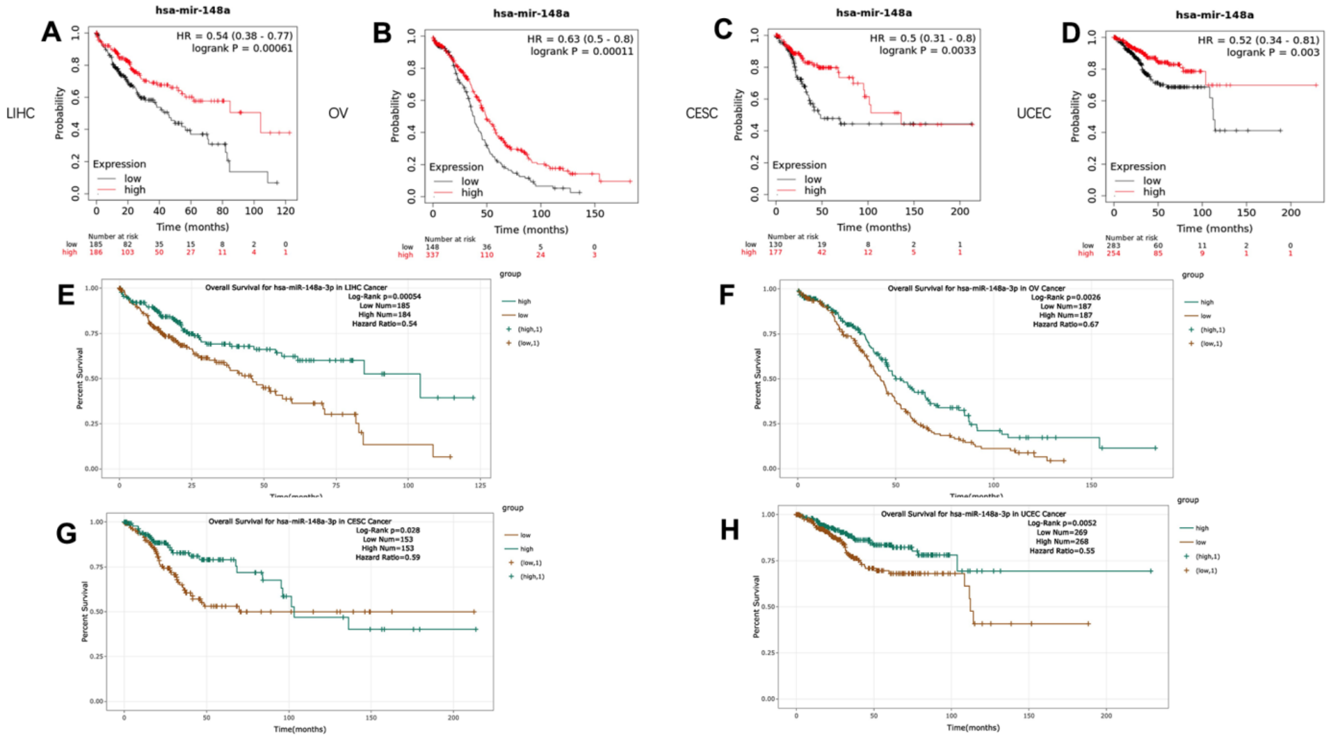

Supplementary Figure 3. Survival analysis of cancer patients with different miR-148a level. Survival curve of LIHC (**A**, **E**), OV (**B**, **F**), CESC (**C**, **G**) and UCEC (**D**, **H**) patients with differential level of miR-148a expression. Analysis was done with Kaplan-Meier Plotter and ENCORI.

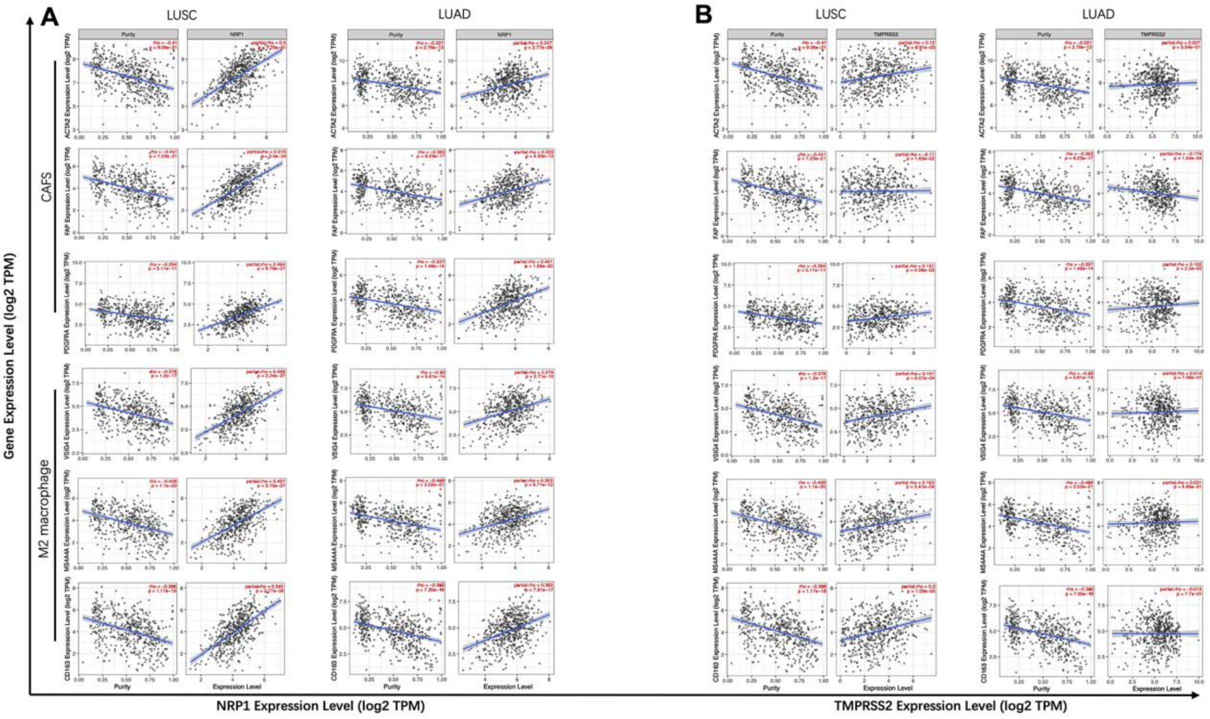

Supplementary Figure 4. Correlation analysis between NRP1(TMPRSS2) and pro-tumorigenic factors. (**A**) Correlation analysis between NRP1 and markers for CAFs and M2 macrophage in LUSC (n=501) and LUAD(n=515). (**B**) Correlation analysis between TMPRSS2 and markers for CAFs and M2 macrophage in LUSC (n=501) and LUAD(n=515). Purity adjusted for all the panels.

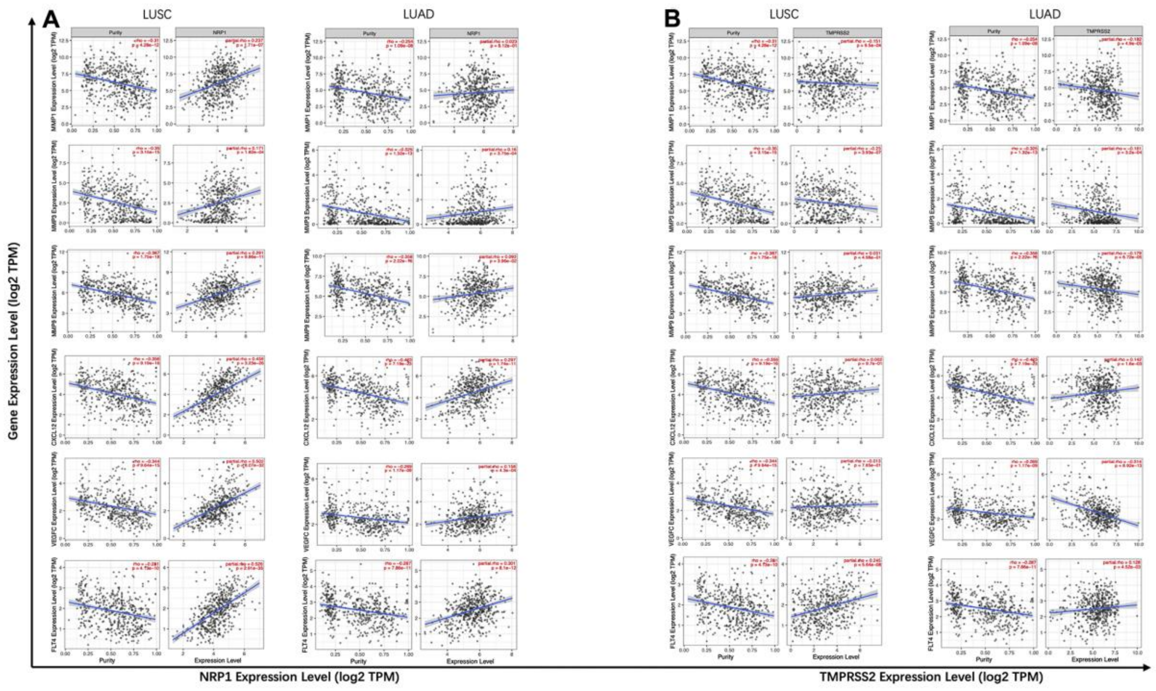

Supplementary Figure 5. Correlation analysis between NRP1(TMPRSS2) and pro-tumorigenic factors (purity adjusted). (**A**) Correlation analysis between NRP1 and MMP1, MMP3, MMP9, VEGFC, FLT4 and CXCL12 in LUSC (n=501) and LUAD(n=515). (**B**) Correlation analysis between TMPRSS2 and MMP1, MMP3, MMP9, VEGFC, FLT4 and CXCL12 in LUSC (n=501) and LUAD(n=515). Purity adjusted for all the panels.
